# Alveolar crystal burden in stone workers with artificial stone silicosis

**DOI:** 10.1111/resp.14229

**Published:** 2022-02-17

**Authors:** Simon H. Apte, Maxine E. Tan, Viviana P. Lutzky, Tharushi A. De Silva, Andreas Fiene, Justin Hundloe, David Deller, Clair Sullivan, Peter T. Bell, Daniel C. Chambers

**Affiliations:** ^1^ Queensland Lung Transplant Service The Prince Charles Hospital Brisbane Queensland; ^2^ Faculty of Medicine The University of Queensland Brisbane Queensland; ^3^ Queensland University of Technology Brisbane Queensland Australia; ^4^ Wesley Hospital Brisbane Queensland Australia; ^5^ Pindara Private Hospital Gold Coast Queensland Australia

**Keywords:** alveolar crystal burden, artificial stone silicosis, inorganic dust, interstitial lung disease, microscopy, pneumoconiosis

## Abstract

**Background and objective:**

An epidemic of silicosis has emerged due to a failure to control risks associated with exposure to high‐silica content respirable dust generated while working with artificial stone products. Methods for quantification of alveolar crystal burden are needed to advance our understanding of the pathobiology of silica‐related lung injury as well as assisting in the diagnosis, clinical management and prognostication of affected workers. The objective of this study was to develop and validate novel methods to quantify alveolar crystal burden in bronchoalveolar lavage (BAL) fluid from patients with artificial stone silicosis.

**Methods:**

New methods to quantify and analyse alveolar crystal in BAL from patients with artificial stone silicosis were developed. Crystals were isolated and counted by microscopy and alveolar crystal burden was calculated using a standard curve generated by titration of respirable α‐Quartz. The utility of the assay was then assessed in 23 patients with artificial stone silicosis.

**Results:**

Alveolar crystal burden was greater in patients with silicosis (0.44 picograms [pg]/cell [0.08–3.49]) compared to patients with other respiratory diagnoses (0.057 pg/cell [0.01–0.34]; *p* < 0.001). Alveolar crystal burden was positively correlated with years of silica exposure (ρ = 0.49, *p* = 0.02) and with decline in diffusing capacity of the lungs for carbon monoxide (ρ = −0.50, *p* = 0.02).

**Conclusion:**

Alveolar crystal burden quantification differentiates patients with silicosis from patients with other respiratory disorders. Furthermore, crystal burden is correlated with the rate of decline in lung function in patients with artificial stone silicosis.

## Graphical abstract

In this study, we develop and validate an assay to quantify alveolar crystal burden in bronchoalveolar lavage fluid from patients with artificial stone silicosis. This assay may be a useful adjunct in the diagnosis of silicosis and assessing response to therapeutic intervention.

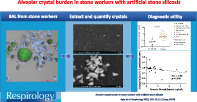

**See related** Editorial

## INTRODUCTION

Silicosis is an ancient respiratory disease caused by inhalation of respirable crystalline silica dust resulting in progressive inflammatory lung injury and fibrosis.^1^ Despite being an entirely preventable condition, rates of silicosis remain unacceptably high in many developing countries,^1^, ^2^ and an alarming resurgence of silicosis in higher‐income countries has prompted concerns that the world is ‘*failing on silicosis*’.^3^ A new epidemic of silicosis has emerged in the stone benchtop manufacturing industry^3^, ^4^, ^5^ due to a failure to recognize and control risks associated with exposure to high‐silica content respirable dust.^6^

Respirable silica crystals deposit within respiratory bronchioles and alveoli and are phagocytosed by alveolar macrophages where they elicit inflammation, reactive oxygen species production, inflammasome activation and ultimately a cascade of parenchymal injury and fibrosis.^7^, ^8^ The use of artificial stone (i.e., conglomerate quartz/engineered stone) products for home benchtops has become increasingly popular over the past two decades. Artificial stone typically contains a crystalline silica content upwards of 90%, far greater than natural stone alternatives,^9^ with cutting, shaping and polishing liberating hazardous respirable particles.^10^ While effective dust control measures exist, the implementation of these measures has been inadequate, resulting in silicosis outbreaks in Australia,^6^, ^11^ the United States,^12^ Europe^13^, ^14^, ^15^ and Asia.^9^, ^16^, ^17^ A recent silicosis outbreak in Australia^11^ has prompted proactive screening of artificial stone workers and has identified a large number of workers with early and more advanced forms of silicosis including progressive massive fibrosis (PMF).^18^, ^19^

Emerging clinical data have begun to challenge our understanding of the pathobiology and natural history of silica‐related lung disease. What was once considered an indolent ‘chronic’ fibrosing lung disease affecting workers with long‐term natural respirable silica dust exposure^1^ appears to manifest a more aggressive and accelerated clinical phenotype in individuals working with artificial stone.^11^, ^12^, ^15^, ^17^, ^20^ Of concern, affected stone workers may progress rapidly to advanced respiratory failure^12^, ^15^, ^16^, ^18^ and in the absence of any existing disease‐modifying therapeutic options, transplant‐free survival remains poor.^16^ Alarmingly, the majority of workers experience progressive disease even after removal from the workplace.^15^ Therefore, early therapeutic interventions aimed at preventing progression to irreversible PMF are urgently needed.

Methods for quantification of alveolar crystal burden are important not only in advancing our understanding of pathobiology, but also for improving the clinical management of affected workers. Quantifying alveolar crystal burden may assist in resolving diagnostic uncertainty, for example, between silicosis and sarcoidosis as well as other diagnoses. In the current study, we develop a simple technique for alveolar crystal quantification in bronchoalveolar lavage (BAL) fluid using microscopy. We then apply this technique to a group of 23 patients with silicosis that were detected through a screening programme in Queensland, Australia. We demonstrate the utility of the technique in differentiating between workers with silicosis, potentially exposed workers with other diagnoses and non‐exposed control subjects. Furthermore, we show that alveolar crystal burden is significantly correlated with exposure history and the rate of decline in diffusing capacity of the lungs for carbon monoxide (DL_CO_).

## METHODS

### Study participants

In Queensland, workers with occupational crystalline silica dust exposure have been subjected to an intensive screening programme for silica‐related lung disease,^21^ often including HRCT of the thorax given superior sensitivity compared to chest x‐ray in early‐stage silicosis.^22^ The diagnosis of silicosis, or an alternative diagnosis, was established at an interstitial lung disease multidisciplinary team meeting (ILD‐MDT) comprised of expert thoracic physicians, radiologists and histopathologists.^23^ As there is often diagnostic confusion, in patients with sarcoidosis, exhaustive investigations were undertaken to exclude a diagnosis of silicosis.

Patients with a diagnosis of silicosis were enrolled in this study if a BAL specimen was available for alveolar crystal examination. BAL was conducted according to international guidelines^24^ by instilling 120 ml of normal saline into either the middle lobe or the anterior segment of the right upper lobe (depending on which was the more affected segment radiologically). No dwell time was allowed, and the lavage was returned using gentle wall suction and was pooled for analysis. Spirometry and DL_CO_ were performed according to the American Thoracic Society (ATS) guidelines^25^ in several laboratories which meet the standards recommended by the Thoracic Society of Australia and New Zealand. Lung function data were collected retrospectively. Rate of change in lung function was modelled by calculating the linear regression slope for each lung function variable versus follow‐up time (in years). Total duration of respirable silica exposure was estimated by calculating the total number of full‐time equivalent years of significant occupational dust exposure based on retrospective review of clinical data. Accelerated silicosis was defined as silicosis that developed within 10 years of initial respiratory crystalline silica exposure.^1^ Chronic silicosis was defined as silicosis that developed beyond 10 years after initial respiratory crystalline silica exposure.^1^ PMF was defined by the development of conglomerate lung nodules of ≥10 mm in diameter.^1^

### Quantification of alveolar crystal burden

The methods for quantification of alveolar crystal burden are provided in Appendix S1 in the Supporting Information. In brief, a known number of cells recovered from BAL fluid were lysed in strong detergent and heated to 95°C to generate a cell extract. The extract was washed several times in water and then centrifuged over a dense sucrose column with the objective of purifying the denser inorganic crystals in the base of the tube. The supernatant was aspirated and the remaining crystals were again washed several times. The crystals were then resuspended in mounting media and viewed in a 96‐well plate on an inverted light microscope. The number of particles in a given field was counted and the mean of three fields was used for each sample. The results were compared to a standard curve generated using similar methods with a known quantity of respirable quartz and then divided by the number of cells used to generate the extract to give crystal load in picograms (pg)/cell.

### Statistical analysis

Patient demographics and clinical data were analysed using non‐parametric statistics unless otherwise stated. Spearman rank‐order correlation coefficient was used to examine correlations between non‐parametric continuous variables of interest. All analyses were performed using the R statistical package (version 3.6.1) and are reported at an alpha of 0.05. Data visualizations were performed using Prism software (GraphPad, San Diego, version 8.4.3).

## RESULTS

Twenty‐three artificial stone workers with a diagnosis of silicosis were enrolled. All patients had a history of significant industrial exposure to dust generated through the processing of artificial stone. Eleven patients had a diagnosis of chronic silicosis, 11 had accelerated silicosis and one patient had chronic obstructive pulmonary disease secondary to silica exposure. Of those patients with accelerated silicosis, three had radiological evidence of established PMF and one patient had extensive reticulation visible on HRCT which fell just short of a diagnosis of PMF at the time of BAL. In addition, one patient with chronic silicosis had a diagnosis of PMF. Two of the four were lifelong non‐smokers, age at diagnosis ranged from 31 to 56 years and duration of full‐time equivalent exposure ranged from 11 to 37 years.

All participants in the silicosis group were male with a median age of 38 years (range 23–64) and a median BMI of 25.0 kg/m^2^ (range 19.4–36.3). The median duration of full‐time work in the stonemasonry industry was 11 years (range 3.5–37). Patients with silicosis had a median % predicted forced expiratory volume over 1 s (FEV_1_) of 90.0% (range 55–139), median % predicted forced vital capacity (FVC) of 99.0% (54–132), median FEV_1_/FVC ratio of 75.0 (range 52–90) and a median % predicted DL_CO_ of 82.0% (13.0, range 54–129). Fifteen patients (65.2%) had a history of cigarette smoking. Fifteen patients (65.2%) had a modified Medical Research Council (mMRC) dyspnoea (mMRC) score of 0, three patients (13.0%) had an mMRC score of 1, four patients (17.4%) had an mMRC score of 2 and one patient had an mMRC score of 3 (4.3%). BAL specimens were acquired from the right upper lobe (*n* = 14), middle lobe (*n* = 8) and left upper lobe (*n* = 1) in patients with silicosis.

A comparator control group comprised eight patients without a diagnosis of silicosis who had undergone alveolar crystal quantification from an available BAL specimen (Table 1). This group included patients with a diagnosis of sarcoidosis (*n* = 3), obstructive sleep apnoea (*n* = 1), asthma (*n* = 1), unclassifiable ILD (*n* = 2) and respiratory bronchiolitis‐ILD (RB‐ILD, *n =* 1). In the comparator group, specimens were collected from the right upper lobe (*n* = 4), middle lobe (*n* = 3) and left upper lobe (*n* = 1).

**TABLE 1:** Demographic and clinical parameters

	Silicosis	Non‐silicosis control	*p*‐value
*n*	23	8	—
Crystal burden (pg/cell), median (range)	0.44 (0.08–3.49)	0.057 (0.01–0.34)	<0.001
Male gender, *n* (%)	23 (100)	7 (87.5)	—
Age, median (range)	38 (23–64)	50.5 (34–62)	0.008
BMI (kg/m^2^), median (range)	25 (19.4–36.3)	31.5 (29.4–32.5)	0.06
Silica exposure years, median (range)	11 (3.5–37.0)	0 (0–15)	0.01
Smoker, *n* (%)	15 (65.2)	3 (37.5)	0.17
FEV_1_% predicted, median (range)	90.0 (55–139)	88.5 (62–96)	0.57
FVC % predicted, median (range)	99.0 (54–132)	91.5 (81–104)	0.16
FEV_1_/FVC ratio, median (range)	75.0 (52–90)	77.0 (51–80)	0.87
DL_CO_ % predicted, median (range)	82.0 (54–129)	82.5 (75–116)	0.77
BAL differential % macrophage, median (range)	81.0 (52–95)	71.0 (28–92)	0.20
BAL differential % lymphocyte, median (range)	8.0 (2–40)	8.0 (4–72)	0.46
BAL differential % neutrophil, median (range)	6.0 (1–28)	6.0 (0–31)	0.90
BAL differential % eosinophil, median (range)	1.0 (0–3)	0 (0–3)	0.40

Abbreviations: BAL, bronchoalveolar lavage; DL_CO_, diffusing capacity of the lungs for carbon monoxide (percent predicted); FEV_1_, forced expiratory volume over 1 s (percent predicted); FVC, forced vital capacity (percent predicted); pg, picogram.

**FIGURE 1:**
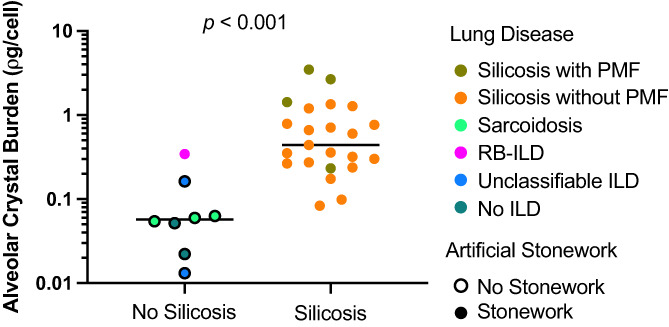
Alveolar crystal burden in silicosis. Crystal quantification in bronchoalveolar lavage fluid differentiates patients with silicosis. The group without silicosis included one patient with a final ILD‐MDT‐endorsed diagnosis of RB‐ILD, who had a 15‐year history of working as a stone worker; two with an ILD‐MDT diagnosis of unclassifiable ILD; three patients with a diagnosis of sarcoidosis; and two patients with no evidence of ILD who were being investigated for chronic cough. One of these had a final diagnosis of cough variant asthma, and the other had a final diagnosis of obstructive sleep apnoea. ILD, interstitial lung disease; ILD‐MDT, ILD multidisciplinary team; PMF, progressive massive fibrosis; RB‐ILD, respiratory bronchiolitis‐ILD

**FIGURE 2:**
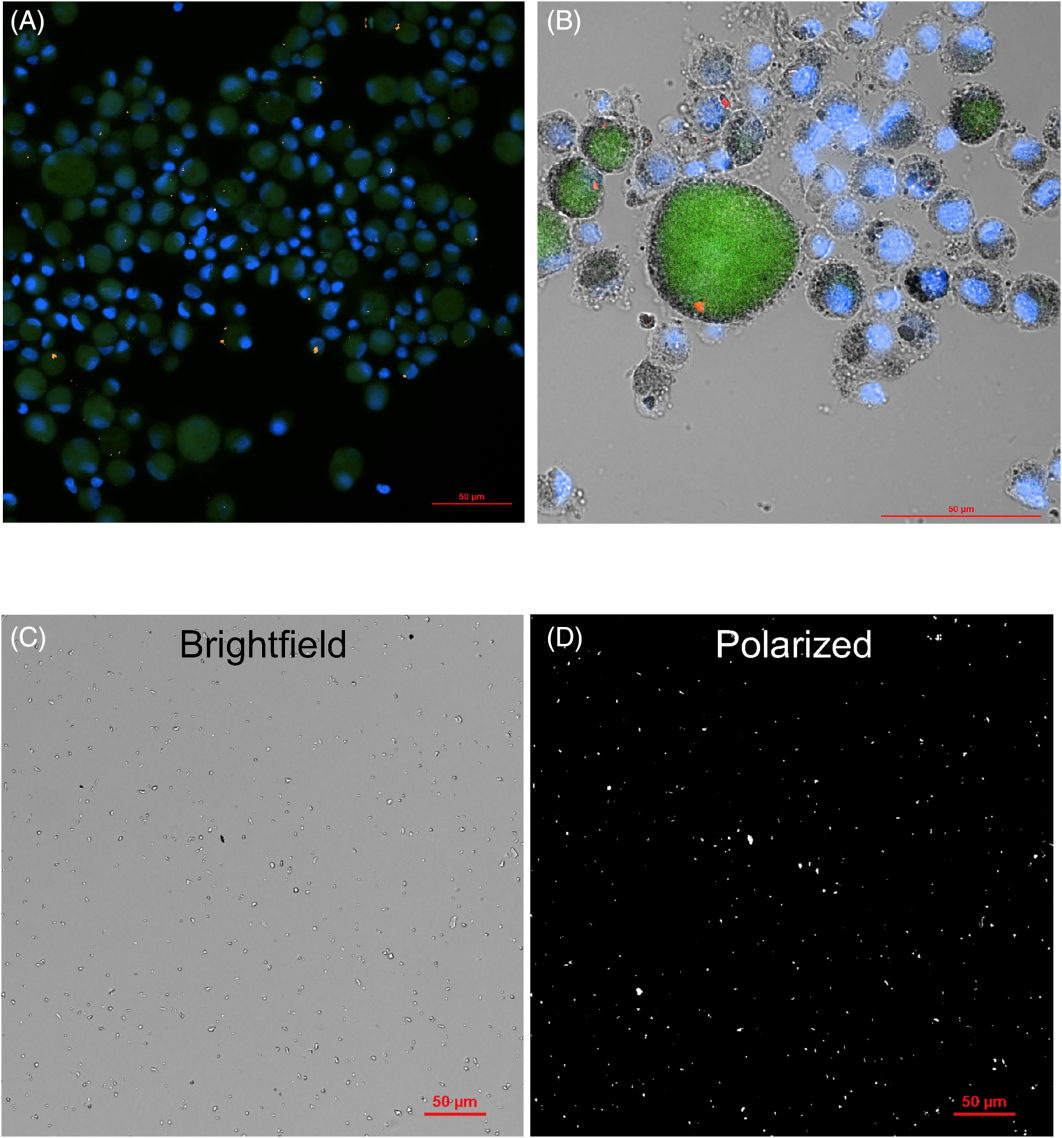
Birefringent crystals are evident within alveolar macrophages of stone workers and can be extracted and purified. Composite microscope images of fixed bronchoalveolar lavage (BAL) fluid collected from a stone worker with silicosis; autofluorescence (green), nucleic acid staining (4',6‐Diamidino‐2‐Phenylindole (DAPI), blue), polarized particles (orange); (A) 30× magnification, (B) 60× magnification and includes a brightfield image. Scale bar is 50 μm. Crystals were extracted and purified from the BAL as described in Appendix S1 in the Supporting Information and mounted in a 96‐well optical plate for imaging on an inverted microscope. (C) Brightfield image of extracted particles (20× magnification) and (D) polarized view of the same frame

**FIGURE 3:**
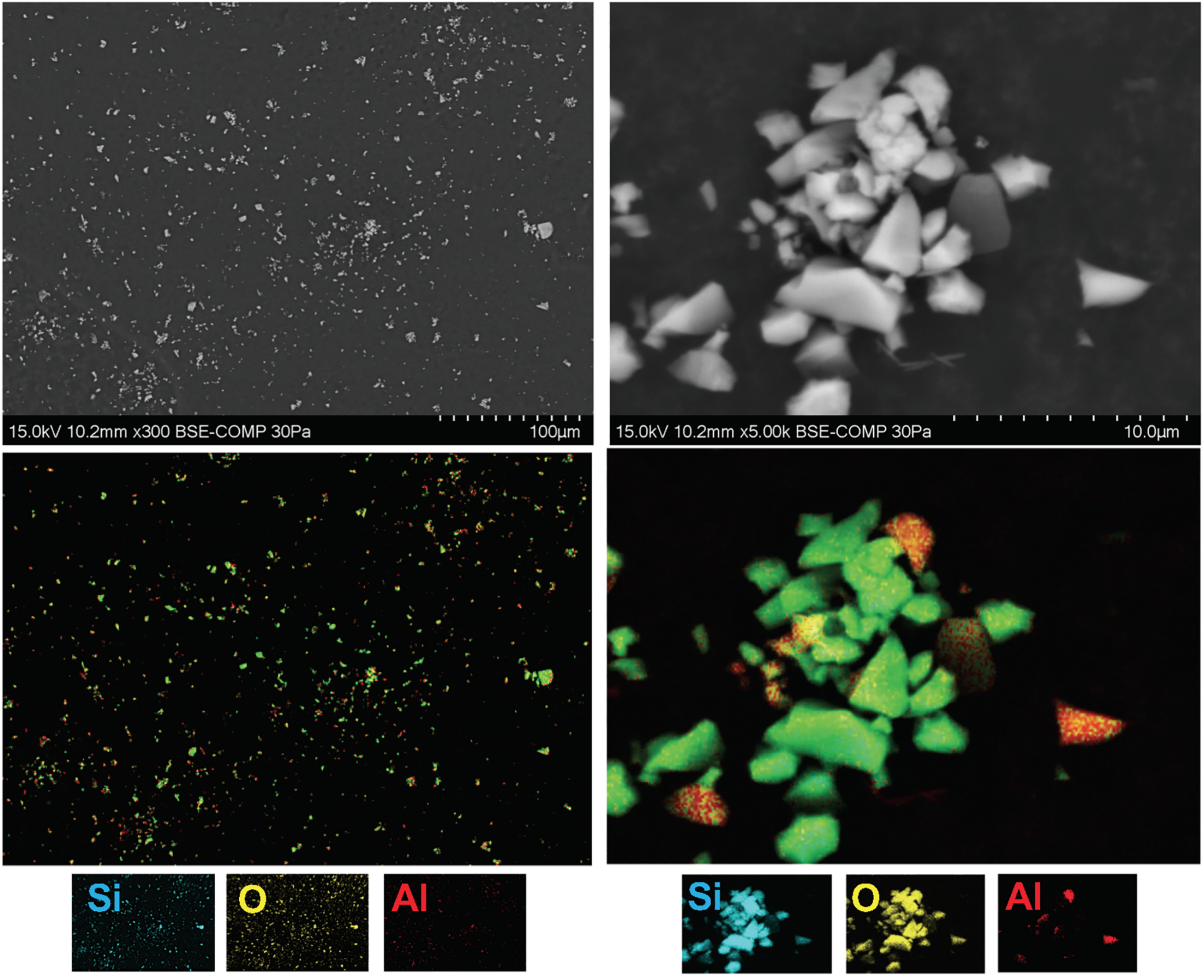
Crystal extracted from the bronchoalveolar lavage fluid (BAL) of stone workers is largely composed of quartz. Crystals were extracted and purified from the BAL of a stone worker as described in the Methods section. The purified crystals were mounted on a carbon stud and assessed by scanning electron microscopy. Top panels show backscatter micrograph (with relevant scale bar). Mid panels show corresponding energy‐dispersive spectroscopy layered maps with individual element maps (Si, O, Al) below. Al, aluminium; O, oxygen; Si, silicon

BAL fluid from patients with silicosis demonstrated greater alveolar crystal burden (median = 0.441, range = 0.083–3.487 pg/cell) compared to the non‐silicosis control group (median = 0.057, range = 0.013–0.344 pg/cell, *p* < 0.001, Figure 1). Brightfield images and polarized images of the same sample confirmed that most particles were birefringent (Figure 2). Electron microscopy was used to determine the atomic makeup of particles. Most particles had corresponding Si and O signals consistent with quartz (silicon dioxide [SiO_2_]) with some particles also containing Al, consistent with aluminosilicates (Figure 3). In the non‐silicosis comparator group, two outliers with a disproportionately high crystal burden were observed. One was a 40‐year‐old male with an ILD‐MDT diagnosis of RB‐ILD, who had a 15‐year history of working as a stone worker and a 20 pack‐year history of cigarette smoking. The other had an ILD‐MDT diagnosis of unclassifiable ILD, with upper zone reticular and sub‐pleural changes with calcification, with no history of occupational exposure to respirable crystalline silica.

**FIGURE 4:**
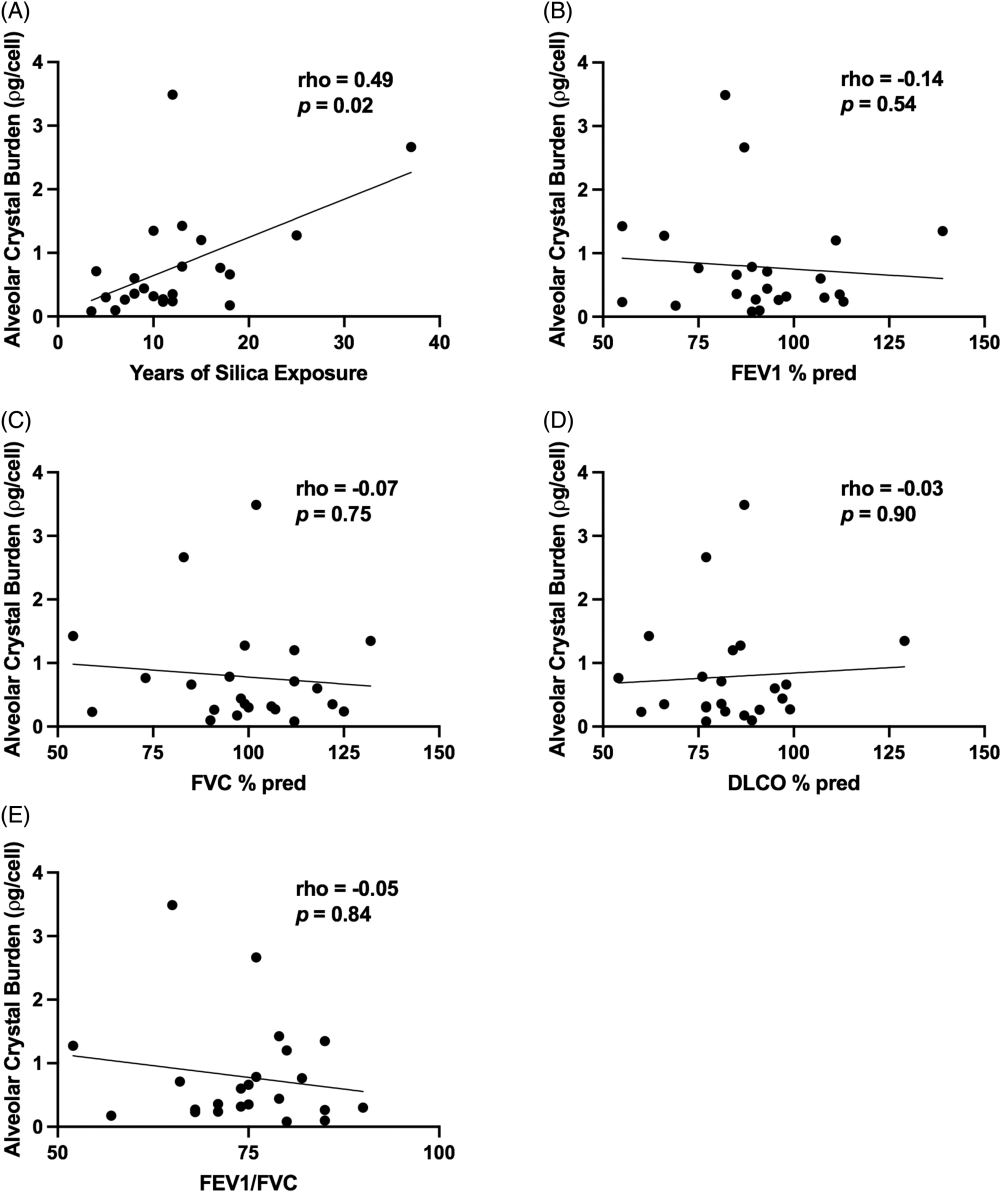
Relationship between baseline clinical variables and alveolar crystal burden. (A) Alveolar crystal burden is related to estimated total duration of full‐time equivalent respirable crystal silica exposure in years. (B–E) Lung function parameters at the time of bronchoscopy were not associated with alveolar crystal burden. DL_CO_, diffusing capacity of the lungs for carbon monoxide (percent predicted); FEV_1_, forced expiratory volume over 1 s (percent predicted); FVC, forced vital capacity (percent predicted)

There was a significant positive correlation between alveolar silica burden and years of full‐time employment in the stone working industry (ρ = 0.49, *p* = 0.018, Figure 4A). There was no significant relationship between alveolar crystal burden and mMRC dyspnoea score (*χ* ^2^ 2.35, df = 3, *p* = 0.50) or spirometric variables at the time of BAL (FEV_1_% predicted, ρ = −0.14, *p* = 0.54; FVC% predicted, ρ = −0.07, *p* = 0.75; FEV_1_/FVC, ρ = −0.05, *p* = 0.84; DL_CO_% predicted, ρ = −0.03, *p* = 0.90, Figure 4B–D).

**FIGURE 5:**
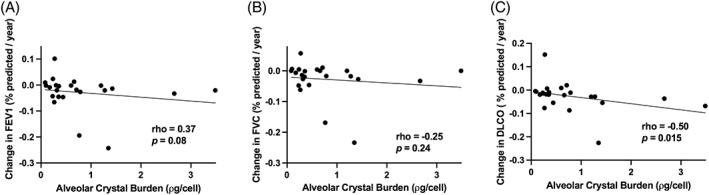
Relationship between alveolar crystal burden and change in lung function over time. Alveolar crystal burden was not related to the rate of change in (A) FEV1 or the rate of change of (B) FVC. (C) There was a significant inverse correlation between silica crystal burden (picograms/cell) and rate of change in DL_CO_. DL_CO_, diffusing capacity of the lungs for carbon monoxide (percent predicted); FEV_1_, forced expiratory volume over 1 s (percent predicted); FVC, forced vital capacity (percent predicted)

There was a significant inverse correlation between crystal burden and change in DL_CO_ over time (ρ = −0.50, *p* = 0.015, Figure 5C). There was no significant relationship between silica crystal burden and rate of decline in other spirometric parameters (FEV_1_, ρ = −0.37, *p* = 0.08, Figure 5A; FVC, ρ = −0.25, *p* = 0.24, Figure 5B; FEV_1_/FVC, ρ = −0.26, *p* = 0.23). Follow‐up time was limited and varied between participants (median follow‐up time 366 days, range 147–1006 days, median number of three spirometry assessments [range 2–5] performed per patient during the follow‐up period).

**FIGURE 6:**
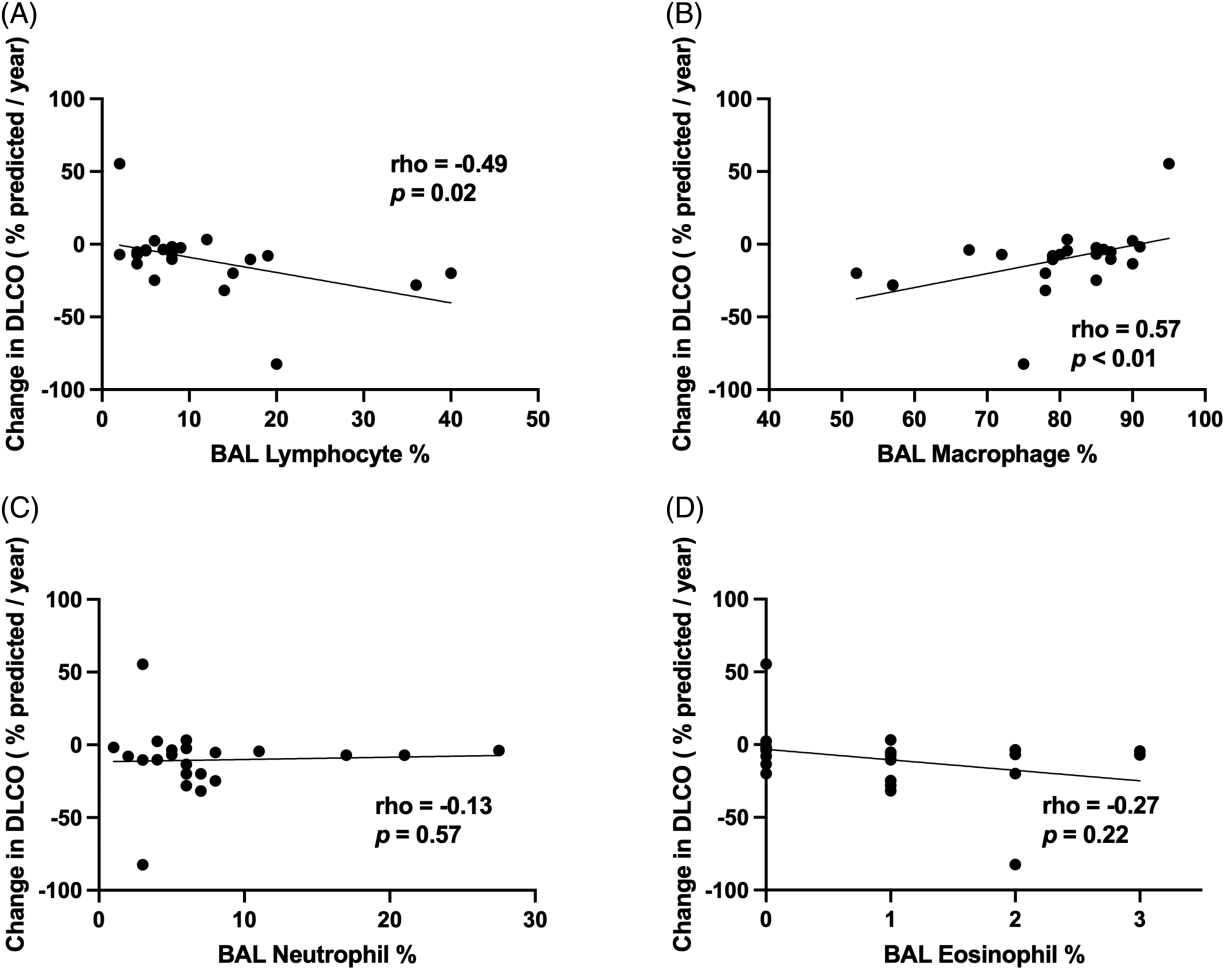
BAL lymphocytosis is associated with the rate of change in DL_CO_. BAL leucocyte differential demonstrated that (A) the proportion of lymphocytes was inversely correlated with the rate of change in DL_CO_ and (B) the proportion of lavage macrophages was positively correlated with rate of change in DL_CO_. Rate of change in DL_CO_ was not associated with (C) the proportion of neutrophils or (D) proportion of eosinophils in BAL fluid, respectively. Note that leucocyte differential from BAL was not available for one patient with silicosis. BAL, bronchoalveolar lavage; DL_CO_, diffusing capacity of the lungs for carbon monoxide (percent predicted)

The proportion of immune cell types present in BAL fluid was not significantly related to the alveolar crystal burden (macrophage, ρ = −0.23, *p* = 0.31; lymphocyte, ρ = 0.28, *p* = 0.21; neutrophil, ρ = −0.05, *p* = 0.82; eosinophil, ρ = 0.01, *p* = 0.97). Post hoc analysis demonstrated that the proportion of lymphocytes in the BAL leucocyte differential was inversely correlated with the rate of DL_CO_ decline (ρ = −0.49, *p* = 0.02, Figure 6). By extension, there was also a positive correlation between the proportion of macrophages and DL_CO_ decline (ρ = 0.57, *p* < 0.01) in the BAL. Rate of change in DL_CO_ was not associated with the proportion of neutrophils (ρ = −0.13, *p* = 0.57) or eosinophils (ρ = −0.27, *p* = 0.22) in BAL fluid.

## DISCUSSION

Australia and other higher‐income countries are witnessing a resurgence of silicosis. This new health crisis calls for the urgent application of modern research methods to understand the disease and inform the development of new therapeutic interventions. Here, we describe methods to quantify crystal burden in BAL, and have demonstrated the feasibility of this approach in 23 patients with silicosis. We have confirmed that the crystalline material is composed of silicates, that crystal burden is markedly elevated in patients with silicosis compared to controls with other disorders and show that BAL crystal burden is related to duration of exposure, lung function decline and BAL lymphocytosis.

Various methods have been proposed to quantitate inorganic dust within BAL. Lusuardi et al. used energy‐dispersive x‐ray microanalysis of BAL samples to show a highly significant difference in the Si/S ratio between occupationally exposed subjects and non‐exposed individuals; however, this method was unable to discriminate levels of exposure within the exposed group.^26^ Moreira et al. describe a method of counting birefringent cell particles in BAL macrophages.^27^ While their method has good utility for the research laboratory as its main requirement is a relatively inexpensive microscope with the ability to polarize light, this method is rather laborious and assesses the percentage of crystal‐containing macrophages but does not account for macrophages with multiple particles, as commonly occurs. Indeed, it is difficult to assess all the crystals within a cell as they may not be visible in the two‐dimensional plane due to sampling error or an inappropriate polarizing angle.

In this study, we describe a simple and rapid technique that can reliably quantify inorganic alveolar crystals within BAL fluid. Polarized light is commonly used to identify crystalline material in cytologic and histopathologic specimens, but importantly many organic crystals are bi‐refringent, while quartz can be weakly birefringent. The methods described herein exclude crystals that are sensitive to heat and detergent, effectively including non‐mineralogic crystalline material such as cholesterol. The method requires no specialized equipment apart from a simple inverted light microscope, common in most laboratories. Our method quantifies all particles within the recovered BAL fluid, so it is not limited to those within macrophages, although in our experience crystals are almost always intracellular. We have demonstrated that most particles recovered by our method are quartz or aluminosilicates. Therefore, we propose that this technique may provide a useful adjunct to assist in arriving at a final diagnosis; assessing the response to interventions, such as removal from the workplace or whole lung lavage; and in further exploring mechanistic inter‐relationships between respirable crystalline silica, inflammatory lung injury and clinical disease.

The diagnosis of accelerated or chronic silicosis is typically made on clinical grounds, with lung biopsy reserved only for situations of significant diagnostic uncertainty. A key component of the diagnostic work‐up is the careful elucidation of a detailed occupational history; however, it is known that the severity of disease can vary markedly amongst individuals with a similar occupational history. Although we found a positive relationship between years working as a stoneworker and alveolar crystal burden, this relationship was by no means perfect. We suggest that alveolar crystal burden, as the end result of a complex interplay between duration and intensity of exposure, composition and size of airborne particles and efficacy of intrapulmonary clearance, may better reflect the risk of developing significant lung disease, representing an important adjunct to the occupational history. In addition to this clinical challenge, differentiating between silicosis and sarcoidosis represents a frequent conundrum.^7^ Complicating matters further, there is substantial epidemiological,^28^ radiological^29^ and histopathological overlap^30^, ^31^ between these disease entities, posing additional diagnostic challenges.^7^ Here, we have shown that alveolar crystal quantification differentiates patients with significant respirable crystalline silica exposure, even in early‐stage asymptomatic disease. Interestingly, the patient in the comparator group with the greatest alveolar crystal burden had an ILD‐MDT‐endorsed diagnosis of RB‐ILD, but also a history of respirable silica dust exposure through processing of artificial stone benchtops without industry‐standard personal protection. It is possible that the inclusion of information about BAL crystal burden may have led to a different ILD‐MDT outcome. Overall, quantifying alveolar crystal burden from BAL samples may provide useful adjunctive information to assist in refining the differential diagnosis without invasive tissue sampling.

Respirable silica crystals deposit within respiratory bronchioles and alveoli resulting in a cascade of parenchymal inflammation, destruction of alveolar architecture and pulmonary fibrosis.^7^, ^8^ We demonstrated a significant correlation between the rate of decline in DL_CO_ and both alveolar crystal burden and BAL lymphocytosis in patients with silicosis related to artificial benchtop manufacturing and fabrication. These results may reveal important mechanistic relationships between alveolar crystal burden, severity of lymphocytic alveolitis and impairment in alveolar gas exchange in patients with silicosis. An important next step will be to determine whether alveolar crystal quantification or BAL lymphocytosis can *predict* future clinical deterioration and requirement for lung transplantation. Predictive models and disease endotyping may also play a role in targeting specialized interventions for those patients at high risk of clinical deterioration during early‐stage disease.

There are several limitations to this study. First, the sample size and follow‐up time in this study were limited and lung function data were obtained retrospectively from the medical records, so the frequency and duration of follow‐up varied. Second, only patients undergoing bronchoscopy and BAL for a clinical indication were included in the comparator group. It would be of great interest to assess alveolar crystal burden in workers who have been exposed to artificial respirable dust but who have no evidence of silicosis. Future work is needed to establish the potential role of this technology in supporting clinical reasoning in the context of diagnostic uncertainty. Third, BAL was performed on lung segments that were most severely affected radiologically; however, because the spatio‐temporal heterogeneity of crystal distribution has not been characterized, it is not clear whether segmental lavage is linearly related to overall lung crystal burden. Fourth, the rate of DL_CO_ decline was modelled using linear regression; however, longer‐term follow‐up may reveal a non‐linear trajectory of deterioration in some patients with silicosis. Finally, our method purifies dense particles from BAL material. Although we have demonstrated using scanning electron microscopy (SEM) that these particulates are almost exclusively SiO_2_, other minerals and metals may be present.

In conclusion, exposure to silica is the leading cause of occupational lung disease worldwide,^2^ but remains an orphan disease. The methods described herein go some way to providing a precision approach which can be deployed during diagnostic work‐up and as a surrogate endpoint to obtain high‐quality evidence for potential treatments where prospective randomized clinical trials using clinical endpoints are unlikely to be practical to perform.

## CONFLICT OF INTEREST

None declared.

## AUTHOR CONTRIBUTION

**Simon H. Apte:** Conceptualization (equal); data curation (equal); investigation (equal); methodology (equal); software (equal); visualization (equal); writing – original draft (equal); writing – review and editing (equal). **Maxine E. Tan:** Data curation (equal); investigation (equal); methodology (equal); project administration (equal); writing – review and editing (equal). **Viviana P. Lutzky:** Data curation (equal); investigation (equal); methodology (equal); writing – review and editing (equal). **Tharushi A. De Silva:** Data curation (equal); investigation (equal); methodology (equal); writing – review and editing (equal). **Andreas Fiene:** Data curation (equal); writing – review and editing (equal). **Justin Hundloe:** Data curation (equal); writing – review and editing (equal). **David Deller:** Data curation (equal); writing – review and editing (equal). **Clair Sullivan:** Data curation (equal); formal analysis (equal); writing – review and editing (equal). **Peter T. Bell:** Data curation (equal); formal analysis (equal); visualization (equal); writing – original draft (equal); writing – review and editing (equal). **Daniel C. Chambers:** Conceptualization (equal); data curation (equal); project administration (equal); resources (equal); supervision (equal); validation (equal); visualization (equal); writing – original draft (equal); writing – review and editing (equal).

## HUMAN ETHICS APPROVAL DECLARATION

The study was approved by The Prince Charles Hospital HREC – approval: HREC/2018/QPCH/44293. All participants provided written informed consent to participate in the study.

## Supporting information

Supporting Information

**Visual Abstract** Alveolar crystal burden in stone workers with artificial stone silicosis

## Data Availability

The data that support the findings of this study are available on request from the corresponding author. The data are not publicly available due to privacy or ethical restrictions.
