# Supplementary material for: Alveolar crystal burden in stone workers with artificial stone silicosis
Source: Respirology. 2022 Feb 17;27(6):437–46. doi: 10.1111/resp.14229 (PMC9307012; doi:10.1111/resp.14229)
Supplement: Supplementary file 1 — Supporting Information [file RESP-27-437-s002.docx]

**SUPPORTING INFORMATION**

**Alveolar crystal burden in stone workers with artificial stone silicosis**

Apte SH^1,2^, Tan ME^1,2^, Lutzky VP^1,2^, De Silva TA^1,3^, Fiene A^1^, Hundloe J^4^, Deller D^5^, Sullivan C^2^, Bell PT^1,2^, Chambers DC^1,2^

^1^ Queensland Lung Transplant Service, The Prince Charles Hospital, Brisbane, Queensland, Australia

^2^ Faculty of Medicine, The University of Queensland, Brisbane, Queensland, Australia

^3^ Queensland University of Technology, Brisbane, Queensland, Australia

^4^ Wesley Hospital, Brisbane, Queensland, Australia

^5^ Pindara Private Hospital, Gold Coast, Queensland, Australia

**Appendix S1- Additional Methods**

*S.1 Quantification of Alveolar Crystal Burden*

*S.1.1 Titration and Counting of Silica Crystals by Microscopy*

A titration was prepared from respirable α-Quartz reference material (NIST 1878a; median particle size 1.6 um, size distribution: 10%< 0.95um, 25%,1.17um, 50%<1.58um, 75%<2.48um, 90%<3.78um) suspended in reverse osmosis water (R.O. H_2_O) in 1.5ml Eppendorf tubes. The final volume in the tube was 100ul to which 100ul of VECTASHIELD^®^ (H-1000, Vector Laboratories) mounting media was added and vigorously mixed. 200ul of the sample was transferred to a 96 well cell imaging plate (Eppendorf, part# 0030741.030) and then centrifuged in three steps: 10RCF (relative centrifugal force) for 1min; 30RCF for 1min; 200RCF for 2min. The wells were then imaged in an inverted light microscope at 20X (Nikon Eclipse TS100) fitted with a camera. Three images (fields) were recorded for each titration as a JPEG file. The files were assessed using ImageJ software to count the particles in each field. A standard curve with high fidelity was generated (Figure S1).

*S.1.2 Preparation and Quantification of Crystal Extract from BAL*

BAL collected as described in the methods was kept on ice and was processed in the laboratory within 1.5 hours of collection. The volume of BAL was noted and centrifuged at 800RCF for 8 minutes (refrigerated to 4ºC). 2 x 1ml tubes of supernatant were collected and stored (-80ºC) and the remaining supernatant was aspirated and the pellet resuspended in 1ml of media. The cells were counted using a haemocytometer with trypan blue discrimination of dead cells. The total number of cells (live and dead) was recorded. 300x10^5^ cells were taken aside, centrifuged, and stored in 350ul RLT lysis buffer (-20ºC) for gene expression analysis if desired. The number of cells taken to prepare the extract varied depending on the total cell number but was normally around 3x10^6^.

The required volume of cells for the extract was centrifuged as above, aspirated and resuspended in a preheated (~95 ºC) lysis buffer mix of 10ml of 10%W/W SDS and 5ml of 1%V/W Tween-20 (buffers made in R.O. H_2_O) in a 50ml tube. The tube was then vortexed at high speed for 30 seconds and then heated in a water bath at 95 ºC for 10min, then vortexed and heated again for another 10 min. Following this the sample was vortexed again and then the tube filled with R.O. H_2_O, centrifuged and aspirated as above, then filled again with R.O. H_2_O, centrifuged and aspirated.

The pellet was then resuspended in 10ml of R.O. H_2_O and vortexed briefly before being underlayed with 10ml of sucrose solution (50%W/W in R.O. H_2_O), and centrifuged at 800RCF for 10min at room temperature with low acceleration and low brake. Following the centrifugation the sample was carefully aspirated in a sweeping motion to remove any debris above and within the sucrose, leaving about 1ml in the bottom of the tube. The sample was then washed twice with 50ml of R.O. H_2_O, centrifuged and aspirated as above leaving approximately 1ml in the tube after the second wash. The sample was then resuspended vigorously and transferred to a 1.5ml Eppendorf tube, centrifuged in a microfuge at full speed for 2min. The supernatant was removed leaving 100ul of sample, to which 100ul of VECTASHIELD^®^ was added, vigorously resuspended then 200ul transferred to 96 well plate for centrifugation and microscopic assessment as follows.

The wells were imaged in an inverted light microscope at 20X (Nikon Eclipse TS100) fitted with a camera. Three images (fields) were recorded for each subject as a JPEG file. To count the particles in each field, the files were assessed using ImageJ software; the JPEG files were converted to 8-bit images followed by threshold adjustment using *Maximum Entropy* setting to allow the particles to be automatically counted using the *Analyze Particles* tool. The average count of 3 frames was compared to the standard curve to give the approximate mass of the sample. This was then divided by the cell number taken to make the extract to give an approximate crystal mass/cell (schematic in Figure S1).

**Figure S1. Development of an assay to quantify crystal burden in BAL samples.** (A) Respirable α-Quartz reference material was weighed and titrated, then suspended in mounting media in an optical 96-well plate to generate a standard curve as described in the Supplemental Methods. (B) Schematic of method to assess crystal burden/cell in BAL material from silicosis patients.


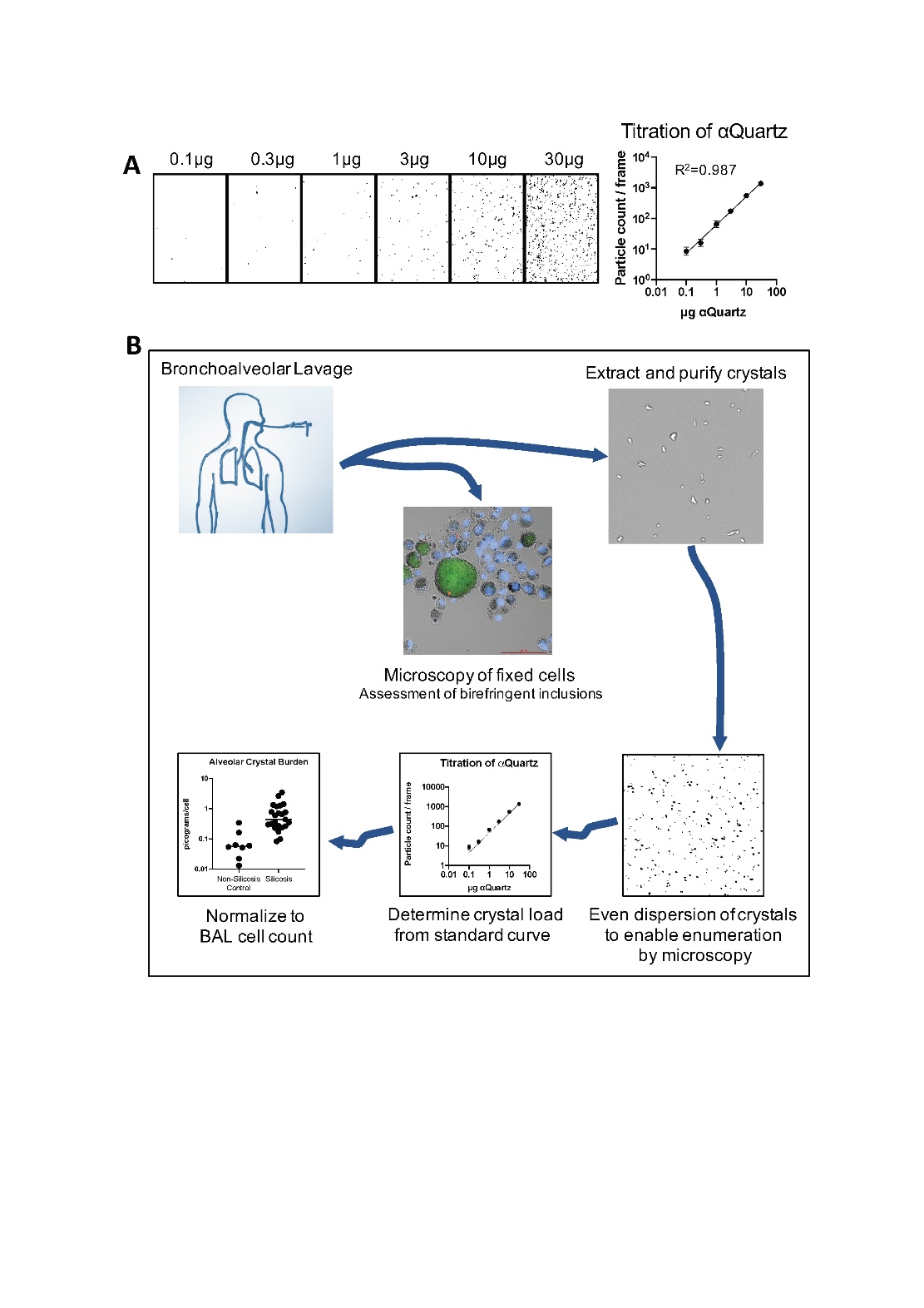


*S.2 Validation of Technique*

*S.2.1 Light Microscopy*

An aliquot of cells from BAL were fixed in fixation buffer (BD Cytofix, part# 554655) and a 5ul drop of cells in fixative were air-dried onto a microscope slide (Menzel-Glaser SUPERFROST^®^ PLUS, Thermo Scientific). The slide was then washed twice with 100% methanol and allowed to dry before rinsing with R.O. H_2_O. The slide was dried and then mounted with a coverslip and 1 drop of antifade with DAPI (ProLong Diamond Antifade with DAPI, Invitrogen) and then sealed with lacquer. The slide was viewed on Nikon Ti2 inverted microscope. Birefringent crystals were detected using polarised light. Crystal extract prepared as described above was imaged on the Nikon Ti2 in an optical 96 well plate (same as for counting); brightfield image and polarised image of same sample shows that most particles are birefringent crystals (Figure 2C & 2D).

*S.2.2 Scanning Electron Microscopy*

Crystals were extracted from BAL cells as described above and washed a further 2 times in full volumes of R.O. H_2_O. The sample was then transferred to 1.5ml Eppendorf tube, centrifuged at full speed for 2 minutes and aspirated leaving about 50ul of fluid. 1 ml of acetone was added to the tube and then centrifuged again then aspirated leaving about 50ul of fluid. The sample was vigorously resuspended and then transferred to a carbon stub mounted for SEM and allowed to air dry. Scanning electron microscopy (SEM) and energy dispersive spectroscopy (EDS) data were acquired using a Hitachi SU3500 Scanning Electron Microscope fitted with an Oxford 50mm^2^ X-Max SDD X-ray detector. The SEM was operated in variable pressure mode at 15 kV (Figure 3).
