# Supplementary material for: Alveolar crystal burden in stone workers with artificial stone silicosis
Source: Respirology. 2022 Feb 17;27(6):437–46. doi: 10.1111/resp.14229 (PMC9307012; doi:10.1111/resp.14229)
Supplement: Supplementary file 2 — Visual Abstract Alveolar crystal burden in stone workers with artificial stone silicosis [file RESP-27-437-s001.pdf]

Alveolar crystal burden in stone workers with artificial stone silicosis

BAL from stone workers

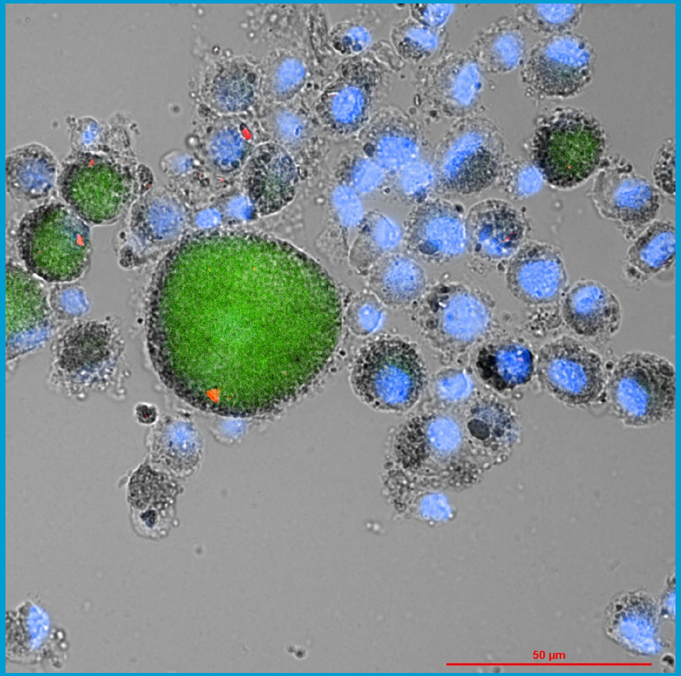

Extract and quantify crystals

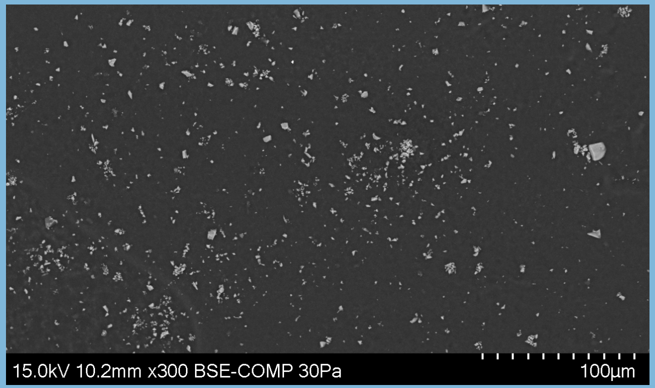

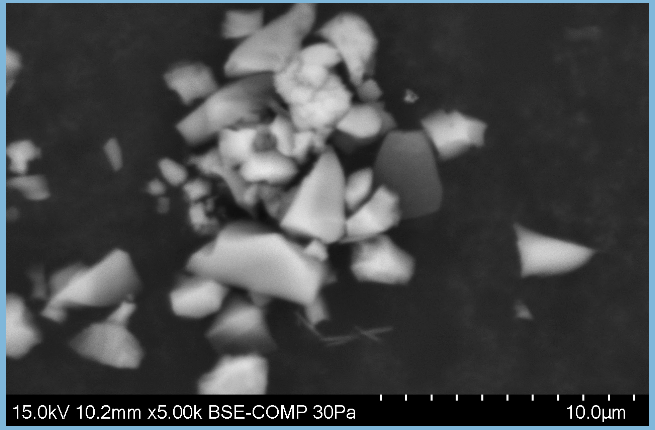

Diagnostic utility

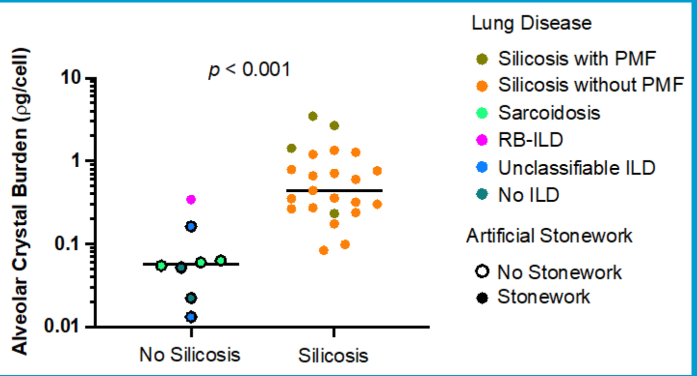

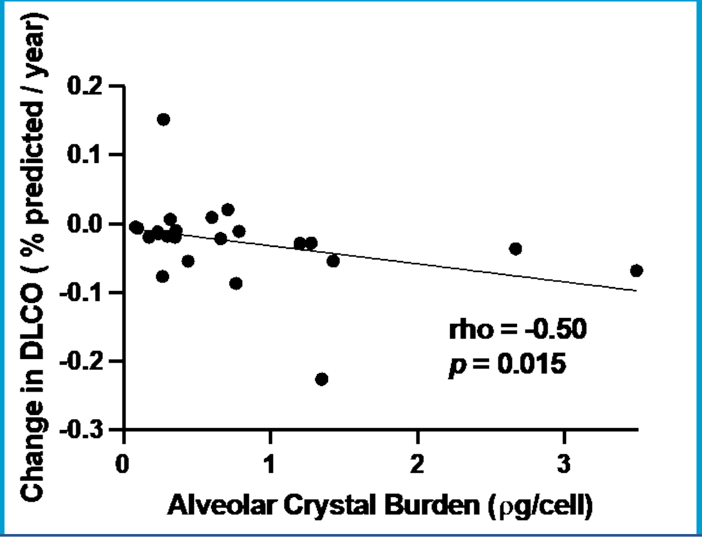

Respirology

Alveolar crystal burden in stone workers with artificial stone silicosis

Apte et al. Respirology 2022; DOI: 10.1111/resp.14229
